# Early Experience With Defensive Antibacterial Coating (DAC®) Hydrogel in High-Risk Orthopaedic Implant Surgery

**DOI:** 10.7759/cureus.114499

**Published:** 2026-08-13

**Authors:** Saikishan Sasi Kumar Menon, Richard Roberts, Shannon Ingram-Walpole, Mohammed Nabeel Shams, David Barlow, Yogesh Joshi, Ibrahim Malek

**Affiliations:** 1 Trauma and Orthopaedics, Wrexham Maelor Hospital, Wrexham, GBR

**Keywords:** antibiotic prophylaxis, arthroplasty, biofilm prevention, dac® hydrogel, high-risk orthopaedic patients, osteosynthesis-associated infection, periprosthetic joint infection, socioeconomic deprivation, surgical site infection

## Abstract

Background: Periprosthetic joint infections (PJIs) after hip and knee arthroplasty and osteosynthesis-associated infections (OAIs) after fracture fixation remain major clinical and economic burdens. High-risk patients with advanced age, multiple comorbidities, and socioeconomic deprivation are particularly vulnerable. Defensive Antibacterial Coating (DAC®) hydrogel (Novagenit S.r.l., Mezzolombardo, Italy) is a fully resorbable hyaluronic acid/poly-D,L-lactide coating that reduces early bacterial adhesion and can act as a carrier for local antibiotic delivery.

Methods: A retrospective cohort study was conducted including patients who underwent orthopaedic arthroplasty or osteosynthesis procedures with intraoperative DAC® application between April 2022 and October 2023. Patients were selected based on clinical factors associated with an increased risk of postoperative infection, including advanced age, multiple comorbidities, the nature of the planned orthopaedic procedure, and consultant surgeon judgement. Demographic, biochemical, and deprivation (Welsh Index of Multiple Deprivation (WIMD)) data were collected. Operative time and perioperative variables were recorded where available. Patients were reviewed clinically and radiographically at six weeks and six months.

Results: Twenty-one patients received DAC®. The mean age was 72.5±15.04 years, American Society of Anesthesiologists (ASA) 2.57±0.74, Charlson Comorbidity Index 5.76±2.34, and deprivation score 6.89±2.37. Procedures included the hip/femur (n=9), knee (n=2), foot and ankle (n=6), and shoulder (n=4). Procedures were further analysed in two groups: arthroplasty (n=6) and osteosynthesis (n=15). The mean operative time was 146.80±32.91 minutes (range: 81-193 minutes). No postoperative implant-related infections were identified during the six-week and six-month follow-up period.

Conclusion: In this real-world high-risk cohort, no postoperative implant-related infections were identified following intraoperative DAC® hydrogel use. Larger controlled studies are required to confirm efficacy and cost-effectiveness.

## Introduction

Periprosthetic joint infections (PJIs) after hip and knee arthroplasty, along with osteosynthesis-associated infections (OAIs) following fracture fixation, remain major clinical and economic challenges worldwide [1,2]. Although infection rates after primary arthroplasty are relatively low at approximately 1-2%, the global rise in orthopaedic procedures means the absolute number of PJIs continues to increase [3,4]. These infections lead to a considerable decrease in patient-reported outcome measures, manifesting as chronic pain, severely diminished joint function, and increased mortality risk. Some evidence indicates that PJIs are associated with a five-year mortality rate as high as 21%, a figure comparable to several common malignancies [5]. Their treatment often involves prolonged hospitalisation, requiring multiple revision surgeries and extended antibiotic regimens, all of which negatively affect patient quality of life [6].

The management of PJIs and OAIs also generates a significant financial strain on global healthcare systems. In England and Wales, revision procedures performed for hip PJI cost, on average, over £41,000 per patient over a five-year period, compared to just over £8,000 for uncomplicated primary arthroplasty [7]. Across the United Kingdom, the annual expense for treating these infections exceeds £300 million, with projections indicating that cumulative expenditure could surpass £1 billion by 2030 [8,9]. Similar financial pressures are evident globally, with annual hospital costs related to PJIs in the United States anticipated to exceed $1.62 billion [10]. 

Several demographic and clinical trends are responsible for the rising incidence of these infections. Increasing surgical demand, driven by population ageing, is expected to result in dramatic growth in primary hip and knee arthroplasty up to 673% and 174%, respectively, between 2005 and 2030 [11]. Patient-related risk factors further increase infection susceptibility: diabetes can double the risk of PJIs [12], and obesity increases absolute infection rates to around 4% [13]. Furthermore, socioeconomic deprivation has been linked to higher postoperative infection rates [14]. Together, these factors highlight a significant need for effective, localised infection-prevention strategies.

The Defensive Antibacterial Coating (DAC®) hydrogel (Novagenit S.r.l., Mezzolombardo, Italy) can be considered one such emerging approach [15-17]. This material, made of hyaluronic acid and poly-D,L-lactide (PDLLA), is a fully resorbable coating and can be applied intraoperatively to implants and adjacent tissues, forming a temporary protective layer. DAC® reduces early bacterial adhesion, which is a significant step in biofilm development [18], and also acts as a vehicle for local antibiotic delivery, maintaining high antimicrobial concentrations at the operative site [19]. A large multicentre prospective study demonstrated that the application of DAC® reduced early postoperative infection rates from 4.8% to 0.5%, supporting its value as an adjunctive infection-prevention strategy [20]. 

Even though clinical results from high-risk and revision cohorts are encouraging [21,22], evidence evaluating DAC® hydrogel in medically complex patients undergoing routine orthopaedic surgery remains limited. This group represents a clinically vulnerable population characterised by multiple comorbidities, impaired wound healing, and heightened susceptibility to infection. Hence, this study aims to describe the real-world use and early clinical outcomes of DAC® hydrogel in high-risk orthopaedic procedures. 

## Materials and methods

**Figure 1:**
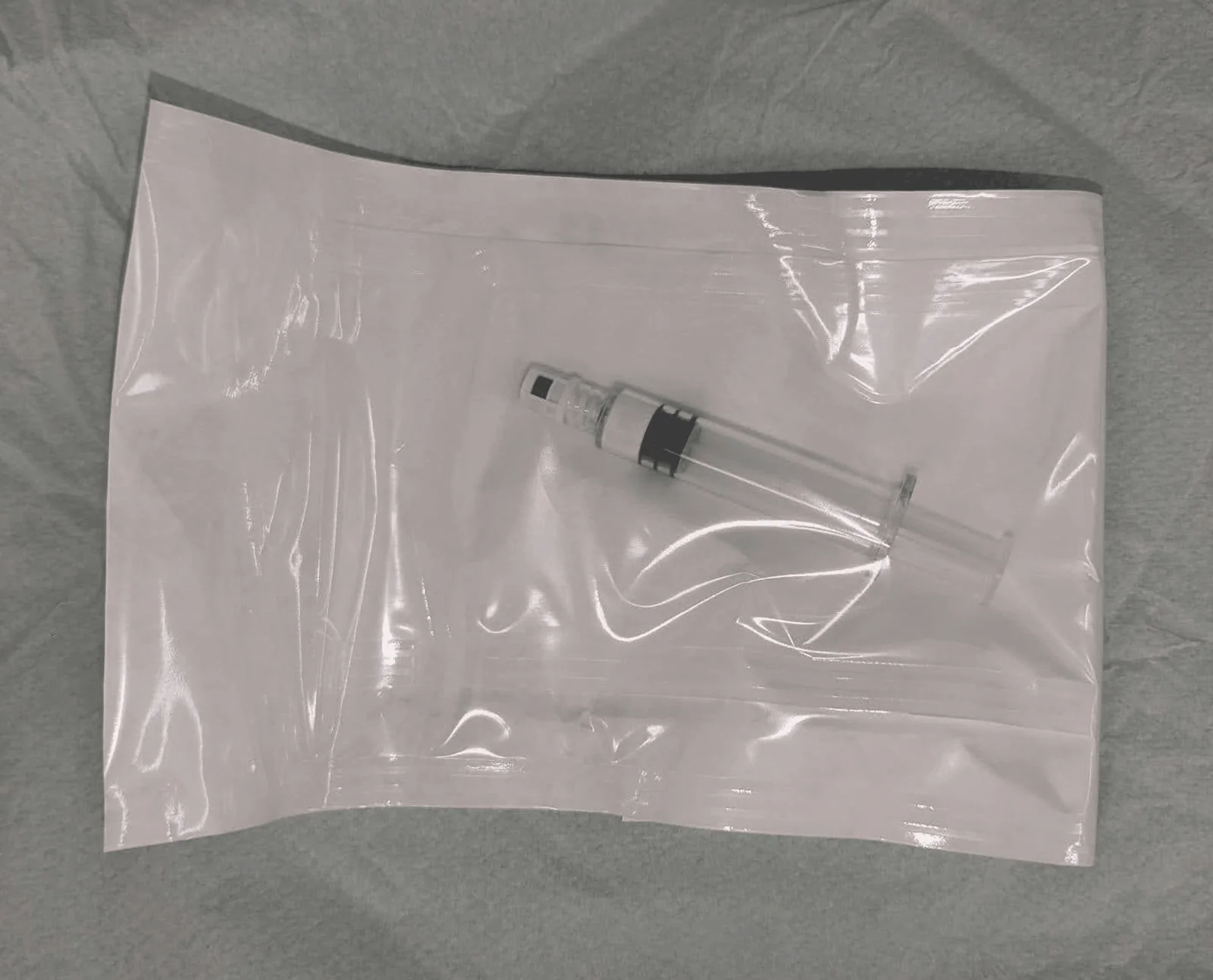
Defensive Antibacterial Coating (DAC®) hydrogel kit in its original sterile peel-pouch packaging. The single-use syringe is packaged to maintain sterility until opened in the operating theatre

A retrospective cohort study was conducted examining patients who underwent orthopaedic arthroplasty or osteosynthesis procedures with the use of DAC® (Figure 1). The study involved reviewing anthropometric and biochemical data, which were extracted from electronic health records and local databases following the use of DAC®. Therefore, as this was an ongoing audit where anonymised data related to the routine clinical care of orthopaedic patients was analysed, ethical approval was not required. 

The primary aim was to evaluate perioperative and long-term infection outcomes. Patients were considered at high risk for postoperative infection based on clinical factors including advanced age, multiple comorbidities, the nature of the planned procedure, and consultant surgeon judgement. In selected cases, the decision to use DAC® hydrogel was made at the discretion of the senior operating surgeon. All patients treated with DAC® were reviewed in outpatient clinics by either a consultant or registrar at both six weeks and six months postoperatively. Radiographic evaluations for signs of infection were conducted, with reports provided by local reporting radiographers and radiologists. Patients were assessed clinically and radiographically at routine follow-up visits, with infection evaluated using the available clinical, microbiological, and radiological findings.

Surgical procedures were categorised into predefined groups, including the hand/foot, wrist, hip, ankle, humerus, elbow, clavicle, and other. Deprivation data were collected from the Welsh Index of Multiple Deprivation (WIMD) for further evaluation between the two groups [23]. The American Society of Anesthesiologists (ASA) Physical Status Classification System [24] and the Charlson Comorbidity Index (CCI) [25] were recorded for each patient as measures of perioperative fitness and comorbidity burden, respectively. For analysis, procedures were further grouped into two broad categories: arthroplasty and osteosynthesis. Outcomes were assessed separately for these groups to improve interpretability. 

Perioperative variables were collected where available, including operative duration, antibiotic use, and surgical technique. Operating time was recorded for all patients and used as a surrogate marker for surgical complexity. Perioperative antibiotic use and surgical approach were recorded where documented. Surgical techniques followed standard orthopaedic practice, and DAC® hydrogel was applied intraoperatively to implant surfaces as per standard practice. Due to the retrospective nature of the study, some perioperative variables were not consistently available across all cases. 

Statistical analysis

Statistical analysis was performed using IBM SPSS Statistics for Windows, Version 28.0 (IBM Corp., Armonk, New York, United States). Results for continuous variables with a normal distribution are presented as mean±standard deviation, and data not following a normal distribution are presented as the median (interquartile range). 

## Results

A total of 21 patients underwent orthopaedic intervention with intraoperative application of DAC® hydrogel between April 2022 and October 2023. The cohort comprised six arthroplasty procedures and 15 osteosynthesis procedures. Baseline demographic, clinical, and perioperative characteristics of the overall cohort and the two procedural groups are summarised in Table 1. Patients undergoing arthroplasty were older than those undergoing osteosynthesis (mean age: 81.18 vs. 69.04 years) and had a shorter mean operative time (129.83 vs. 153.60 minutes).

**Table 1:** Baseline characteristics of patients who underwent procedures where DAC® was used intraoperatively WBC: white blood cell count; ASA: American Society of Anesthesiologists Physical Status Classification; CCI: Charlson Comorbidity Index Social deprivation score was assessed using the Welsh Index of Multiple Deprivation (WIMD).

Variable	Arthroplasty (n=6)	Osteosynthesis (n=15)	Overall (n=21)
Age (years)	81.18± 8.21	69.04±15.94	72.5±15.04
Average length of stay (days)	9.33±8.73	12.26±13.87	11.42±12.47
WBC prior to discharge	7.5±1.25	8.41±2.17	8.10±1.91
ASA	2.5±0.83	2.6±0.73	2.57±0.74
CCI	5.83±0.75	5.73±2.76	5.76±2.34
Social deprivation score	7.5±2.50	6.61±2.36	6.89±2.37
Operative time (minutes)	129.83±32.40 (range: 81-161 )	153.6±31.61 (range: 85-193)	146.80±32.91 (range: 81-193)

The most common procedures were hip or femur surgery (n=9), followed by foot (n=6), shoulder (n=4), and knee (n=2) procedures. Perioperative antibiotic data were available for a subset of patients. Surgical approaches included posterior (hip), deltopectoral (shoulder), medial parapatellar (knee), and lateral approaches for fracture fixation. DAC® hydrogel was applied intraoperatively to implant surfaces according to standard practice.

Postoperative clinical outcomes are summarised in Table 2. No postoperative implant-related infections were identified during either the six-week or the six-month follow-up periods. The exact 95% CI for the observed infection rate, calculated using the Clopper-Pearson method, was 0-16.1% [25]. Two patients died after the completion of the six-month follow-up period from causes unrelated to infection.

**Table 2:** Postoperative clinical outcomes

Outcome	Overall (n=21)
Implant-related infection at 6 weeks	0 (0%)
Implant-related infection at 6 months	0 (0%)
Postoperative radiographs	Satisfactory in all cases
Exact 95% CI for infection rate	0–16.1%
Deaths after the completion of the 6-month follow-up (unrelated to infection)	2 (9.5%)

## Discussion

This study demonstrates that the intraoperative application of the DAC® hydrogel may be associated with reduced postoperative infection rates among high-risk patients undergoing complex orthopaedic surgeries. In our cohort of 21 patients treated with DAC® hydrogel, there were no infections at both six-week and six-month follow-ups. Procedures in this study included both arthroplasty (n=6) and osteosynthesis (n=15). Outcomes have been presented separately for these groups to improve clarity, although infection risk differs between these procedure types. 

Important perioperative factors such as antibiotic use, surgical technique, and operative duration may influence infection risk. In this study, operative time was available for all patients and varied across cases, reflecting differences in surgical complexity. Where documented, antibiotic prophylaxis and surgical approaches were consistent with standard orthopaedic practice; however, these variables were not uniformly recorded. 

Patient comorbidities and surgical infection risk 

The association between patient comorbidities and increased postoperative infection risk is well-established. Conditions such as diabetes mellitus (which increases PJI risk by 1.7-2 times) [12] and obesity (associated with infection rates as high as 4%) [26] are recognised independent risk factors for PJIs. The CCI is a validated tool for quantifying this burden, where higher scores correlate with increased rates of morbidity and mortality. Existing evidence confirms that elevated CCI scores are recognised as independent, highly predictive factors for surgical site infection (SSI) and PJI [27]. Furthermore, specific comorbidities found in these high-CCI patients are strongly correlated with poor treatment outcomes and increased risk of failure if an infection were to occur [28]. 

However, evidence demonstrates that DAC® hydrogel can reduce infection rates in patients undergoing complex procedures like revision arthroplasty, often associated with higher risk and multiple comorbidities [16]. 

A 2025 matched-cohort study of high-risk revision hip arthroplasty reported lower PJI incidence in the DAC-coated group (1/119) compared with controls (5/119) [22]. 

Our findings correlate with this evidence. The mean CCI in our DAC-treated group was 5.76(±2.34), indicating a very-high-risk patient cohort with multiple existing conditions. Despite this inherent susceptibility to infection, we observed a 0% infection rate in the DAC® group. This result suggests that DAC® hydrogel may have potential as an adjunctive strategy for infection prevention in patients with a high comorbidity burden. However, given the observational design and absence of a comparator group, larger prospective comparative studies are required to determine its effectiveness.

Advanced age and surgical infection risk 

Advanced age is a well-recognised independent risk factor for SSI, with large surveillance studies showing significantly higher SSI rates in patients aged ≥65 years undergoing hip and knee arthroplasty [29]. The use of DAC® hydrogel in high-risk and complex arthroplasty (including revision cases) has been associated with reduced overall infection and complication rates in mixed-age cohorts, though data stratified by elderly patients remain scarce [22].

Our study focused on a distinctly older patient population, with a high mean age of 72.5 years (±15.04) in the DAC-treated group. The observed 0% infection rate in this elderly, high-risk cohort further substantiates the potential of the DAC® hydrogel to provide a protective effect where age-related physiological vulnerabilities would typically increase infection susceptibility. These findings support further evaluation of DAC® hydrogel as an adjunct in high-risk geriatric orthopaedic procedures.

Socioeconomic deprivation 

The DAC-treated group had a social deprivation score of 6.89, indicating that the group was more deprived than average for the Welsh population and therefore at greater socioeconomic disadvantage. Socioeconomic deprivation has been identified as an independent risk factor for adverse postoperative outcomes including higher rates of SSIs and complications after orthopaedic and other surgical procedures [30]. 

Although no implant-related infections were observed in this subgroup, the observational design precludes conclusions regarding the independent effect of DAC® hydrogel. Further comparative studies are required.

Limitations 

There are limitations to consider associated with this study. The sample size is relatively small (n=21), and the observational design may introduce biases. There was no randomisation or blinding, and potential confounding factors could influence the results. Patient selection was based on recognised clinical risk factors and surgeon judgement, which may introduce selection bias. The follow-up period was limited to six months, which may not capture late-onset infections. This was constrained by the introduction of DAC® hydrogel in our unit in 2022, and longer follow-up is required to assess long-term outcomes. 

The absence of a control group limits the ability to determine the specific contribution of DAC® hydrogel to the observed outcomes. Further prospective studies with appropriate comparator groups are needed. A comparable historical cohort of similar high-risk patients treated without DAC® hydrogel was not available for analysis, limiting direct comparison of outcomes. The inclusion of both arthroplasty and osteosynthesis procedures introduces heterogeneity, although results have been presented separately to improve interpretability. Detailed perioperative variables, including antibiotic protocols, surgical technique, and wound care practices, were not consistently documented due to the retrospective design and may act as confounding factors. Future prospective randomised studies are required to minimise these confounding factors.

Although no postoperative implant-related infections were identified, this finding should be interpreted with caution given the small sample size and absence of a comparator group. 

Future directions 

Future research should focus on larger, randomised controlled trials to validate the efficacy of DAC® hydrogel in preventing infections across diverse patient populations. Long-term studies are needed to assess the durability of the hydrogel's protective effects and monitor for any potential adverse reactions. Although DAC® hydrogel represents an additional implant-related cost, this may be offset by the substantial clinical and economic burden associated with implant-related infections. Formal cost-effectiveness studies are required before routine use in all patients can be recommended.

Overall, these findings demonstrate favourable early clinical outcomes and support further prospective evaluation of DAC® hydrogel in high-risk orthopaedic patients.

## Conclusions

This retrospective cohort study indicates that the intraoperative use of the DAC® hydrogel may reduce postoperative infection rates in high-risk patients undergoing complex orthopaedic surgeries. Despite the DAC-treated group having a higher mean age, greater CCI, and increased socioeconomic deprivation, all factors associated with elevated infection risk, no postoperative implant-related infections were identified at both six-week and six-month follow-ups.

These findings suggest that DAC® hydrogel may help mitigate infection risks even among vulnerable populations with multiple comorbidities. Incorporating DAC® hydrogel into surgical protocols could enhance patient outcomes and alleviate the clinical and economic burdens of PJIs. However, the study's limitations, including its small sample size and observational design, necessitate caution. Future randomised controlled trials with larger patient cohorts and extended follow-up are essential to validate these results and determine the long-term efficacy and cost-effectiveness of DAC® hydrogel use.
